# Enriched Environment Promoted Cognitive Function *via* Bilateral Synaptic Remodeling After Cerebral Ischemia

**DOI:** 10.3389/fneur.2019.01189

**Published:** 2019-11-12

**Authors:** Chuanjie Wang, Qun Zhang, Kewei Yu, Xueyan Shen, Yi Wu, Junfa Wu

**Affiliations:** ^1^Department of Rehabilitation Medicine, Huashan Hospital, Fudan University, Shanghai, China; ^2^Department of Rehabilitation Medicine, Jinshan Hospital, Fudan University, Shanghai, China; ^3^Department of Rehabilitation Medicine, Huashan Hospital, Fudan University, Shanghai, China

**Keywords:** bilateral hemispheres, cognition, enriched environment, middle cerebral artery occlusion, synapse

## Abstract

Ischemic stroke is the second leading cause of death worldwide. Ischemia-induced cognitive dysfunction may result in a poor quality of life. Synaptic plasticity plays a key role in cognition promotion. An enriched environment (EE), which can attenuate cognitive deficits in chronic cerebral hypoperfusion, has been shown to facilitate synaptic plasticity. However, the effect of EE on synaptic plasticity in bilateral cerebral hemispheres in stroke remains unclear. This study used a permanent middle cerebral artery occlusion mouse model, which was divided into standard housing and EE groups. The Morris water maze test was performed to detect the cognitive function. Electron microscopy was used to determine the synapse numbers. The expression of SYN and GAP-43 was then quantified by immunofluorescence staining and Western blot analysis. Compared with the standard housing, EE promoted the cognitive function recovery in the mice with stroke. Moreover, EE increased the synapse numbers and the expression of SYN and GAP-43 in both the ipsilateral and contralateral hemispheres (*P* < 0.05). A further correlation analysis revealed a positive correlation between the cognitive function outcomes and the relative expression of GAP-43 and SYN. Furthermore, the correlation of the expression of GAP-43 and SYN with cognitive function was higher in the contralateral brain than in the ipsilateral brain. In conclusion, an EE may promote cognitive function *via* bilateral synaptic remodeling after cerebral ischemia. Also, the contralateral brain may play an important role in the recovery of cognitive function.

## Introduction

Ischemic stroke is the second leading cause of disability and death worldwide (1). Cognitive dysfunction can be induced by ischemia, seriously lowering the quality of life (2). The data showed that about 10% of patients suffered from prestroke dementia, 10% progressed to poststroke dementia soon after their first stroke, and more than one third exhibited dementia after recurrent stroke (3, 4). However, the therapies for stroke-related cognition decline are limited. Previous studies illustrated that rehabilitation therapies could protect the brain against neurodegenerative disorders. The mechanisms involved in the beneficial effects on cognition promotion may be correlated with synaptic plasticity (5, 6). Since cerebral ischemia suppresses the expression of synaptic markers such as synaptophysin and PSD-95, the rehabilitation that can enhance synaptic plasticity represents a potential therapeutic tool for enhancing adaptive memories and contrasting the onset and progression of disorders of cognitive functions after stroke (7, 8).

Environmental enrichment (EE) was established to study environment-induced neural plasticity, which demonstrated to improve cognitive impairment in humans (9). EE provide complex ways to stimulate mice with ischemic stroke, increasing social interaction and physical exercise (10, 11). EE paradigm has been reported to improve functional outcomes after focal cerebral ischemia (9, 12). However, most studies focused on angiogenesis and synaptic plasticity in the ipsilateral hemisphere (13–16). However, the role of EE in improving cognitive impairment in the contralateral hippocampus after stroke has been rarely reported.

The bilateral hemisphere often reacts differently to brain disorders, and each hemisphere has its separate function in normal brain activity. Anatomical connections in uninjured brain regions may change in response to a subcortical stroke (17, 18). The contralateral plasticity was observed in unilateral cerebral ischemia models (19). Thus, neurophysiological remodeling in both ipsilateral and contralateral hemispheres might affect the cognitive function recovery after stroke rehabilitation. Studies exploring the bilateral hemisphere competition are important for stroke treatment. However, the roles of bilateral cerebral hemispheres in functional recovery after cerebral ischemia are still unclear.

The present study aimed to test: (1) whether EE could promote cognitive recovery after ischemic stroke in mice; (2) whether EE could influence the synaptic plasticity of bilateral hemispheres after ischemic stroke in mice; and (3) which brain hemisphere plays a more important role in the recovery of cognitive function after EE intervention.

## Materials and Methods

### Experimental Design

**Figure 1:**
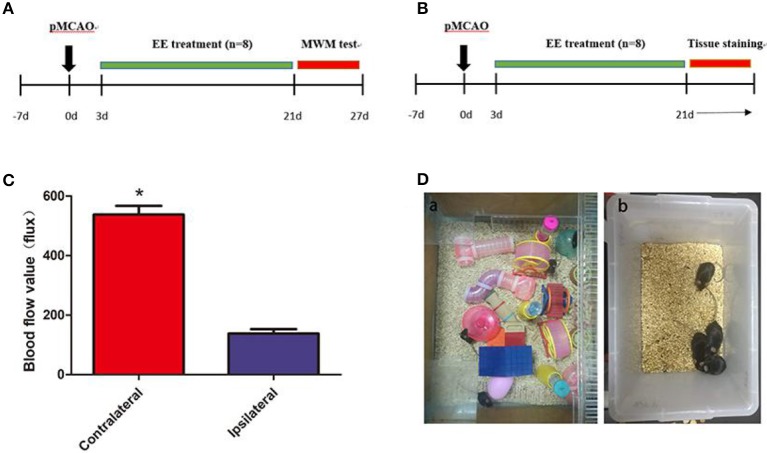
A diagram of the experimental design and brain blood flow after permanent middle cerebral artery occlusion (pMCAO). **(A)** A diagram of the experimental design. The mice underwent pMCAO on day 0. Enriched environment (EE) treatment lasted 3–21 days after pMCAO. The Morris water maze (MWM) test was performed 22–27 days after pMCAO. **(B)** Mouse brain was collected for Western blot analysis, immunohistochemistry, and transmission electron microscopy 21 days after pMCAO. **(C)** Representation of contralateral and ipsilateral blood flow after pMCAO (when the cerebral artery was occluded). *P < 0.05, *n* = 8 per group. **(D)** Pictures demonstrated EE **(a)** and standard housing (SH) **(b)**.

Male, clean C57BL/6 mice (*n* = 72), weighing 25–28 g, were provided by Jie Si Jie Lab Animal Ltd., Shanghai, China. The mice underwent permanent middle cerebral artery occlusion (pMCAO). They were randomly divided into three groups: (1) sham (*n* = 16); (2) standard housing (SH) treated (*n* = 28); and (3) EE treated (*n* = 28) (Figures 1A,B). In the present study, not all of the structural and biochemical evaluations were performed on the same animals. Specifically, the experiment was divided into three sequences (Table 1, Sequences A and B, Data C). Sequence A: The brain tissues of the mice (n = 8 in each group) were used for Western blotting and transmission electron microscopy. Sequence B: The brain tissues of the mice (*n* = 8 in each group) were used for immunofluorescence. Data C: A total of 24 mice (*n* = 8 per group) specifically underwent behavioral test, and the water maze data were used in this manuscript, while the motor-related data were present in one of our other studies. The Institutional Animal Care and Use Committee of Fudan University approved the experimental protocols (No. 20160858A232). All efforts were made to minimize animal suffering.

**Table 1:** The number of animals included in each group for different evaluations.

**Sequence**	**Sham (*n*)**	**Sham (*n*)**	**Sham (*n*)**
Sequence A (WB+TEM)	8	8	8
Sequence B (IF)	8	8	8
Data C (MWM)	8	8	8

*WB, Western blot; TEM, transmission electron microscopy; IF, immunofluorescence; MWM, Morris water maze*.

### Surgical Procedures of pMCAO

The mice were anesthetized with ketamine (100 mg/kg) and xylazine (10 mg/kg) intraperitoneally before the surgery, and the body temperature was maintained at 37°C ± 0.5 using a heating pad (RWD Life Science, Shenzhen, China). A midline incision was made on the neck. A silicone-coated 6-0 suture (Dermalon, 1756-31, OH, USA) was inserted from the external carotid artery stump to the internal carotid artery to induce pMCAO. The distance from the bifurcation to the ostium of the MCA was 8 ± 0.5 mm. The successful occlusion of MCA was verified using a laser Doppler flowmeter (Moor Instruments, Devon, UK) as a decline in the regional blood flow by more than 70% compared with the contralateral hemisphere (Figure 1C). The mortality in the present study was about 20%. The filament in the sham mice was removed immediately after insertion into the MCA (20). Then, the mice were housed in SH or EE 3 days after the surgery (Figure 1D).

### EE and SH Paradigm

The home cage for the EE group was 65 cm wide × 75 cm long × 25 cm high and contained climbing ladders, plastic tubes and tunnels, running wheels, and small boxes (Figure 1Da). At the same time, in order to simulate the rehabilitation environment in the clinical ward, we put two normal mice into the enriched environment (EE) group (eight EE group and two normal mice) because other than ward mates, stroke patients may also come in contact with healthy persons who may offer them different social stimulus. The objects were changed every 3 days to maintain the novelty in the environment. SH and Sham mice were housed in groups of four in standard accommodation (21 cm wide × 27 cm long × 16 cm high) with no objects (Figure 1Db).

### Morris Water Maze (MWM) Test

The MWM test was performed to evaluate the ability of spatial learning and memory of the mice. The test was performed as described in the protocols (21). The experiments included two assessments: spatial acquisition trials and probe tests. All trials were performed in a quiet environment. The swimming pool was a circular tank, 122 cm in diameter, virtually divided into four equal quadrants. The water temperature was approximately 20–22°C. The 10 cm^2^ hidden circular platform was located in the quadrant of NE and submerged 0.5–1.0 cm below the water surface.

The spatial acquisition trials were conducted over 5 consecutive days. Each day had four trials. The start locations were SE, S, NW, and W, as shown in Table 2. If the mouse failed to reach the platform within 1 min, it was guided to the platform. After the animal reached the escape platform, it was allowed to remain there for 15 s before the next trial. The escape latency was defined as the time that the animal spent in reaching the escape platform. The probe test was conducted on day 6. The escape platform was removed from the pool, and the mice were allowed to swim for 60 s. The time and travel distance spent in the target quadrant and the number of times that the animal crossed the zone of the former platform were recorded. All the swim paths were monitored using image detectors and analyzed using the ANY-maze software (ANY-maze, Stoelting Co., IL, USA).

**Table 2:** Start positions for the spatial and probe tests of Morris water maze.

**Day**	**Trial 1**	**Trial 2**	**Trial 3**	**Trial 4**
1	S	W	NW	SE
2	NW	S	SE	W
3	SE	NW	W	S
4	W	SE	S	NW
5	S	NW	W	SE
6 (Probe)	SW			

*NW, Northwest; S, south; SE, southeast; W, west*.

### Transmission Electron Microscopy

Twenty-one days after pMCAO, all mice were anesthetized with an overdose of chloral hydrate and intracardially perfused with 0.9% saline, followed by 20 ml of 4% paraformaldehyde. The brains of the mice were exposed immediately, and samples (approximately 1 mm^3^) were taken from hippocampal CA3. The tissues were postfixed with 2.5% glutaraldehyde overnight. Brain ultrathin sections (~65-nm thick) were used for collecting electron microscopy images. The synapse number was calculated as previously described (22). The estimations were made using the size-frequency method. The formula was as follows: the number of synapses per unit volume = the number of synaptic junctions per unit area of an electron micrograph/the mean length of densities associated with the synaptic junctions. Axo-axonal synapses were not included in the analysis (23).

### The Specimen Handling for Transmission Electron Microscopy

After the mice were executed, we exposed their brains immediately. The bilateral cerebral cortex was gently separated along the coronal sulcus of the brain with anatomical tweezers to expose the bilateral hippocampus beneath the cortex. The hippocampus tissue was then transferred to the optical microscope for the location of hippocampal CA3 area under 20-fold microscope. About 1 mm^3^ of the hippocampal CA3 area was clamped with anatomical tweezers for further electron microscope analysis, while the rest of the hippocampal tissues were stored at −80°C refrigerator for Western blot.

For electron microscope analysis, the tissues were fixed overnight with 2.5% glutaraldehyde in −20°C refrigerator and then rinsed with PBS and soaked in osmium tetroxide. After dehydration in acetone, specimens were embedded in epoxide resin, and 65-nm-thick sections were prepared. After that, the 65-nm-thick sections were put onto copper grids and stained with uranyl acetate followed by lead citrate (24).

### The Synapse Calculation Method and Electron Microscope Analysis

The formula is N_**V**_ = N_**A**_/d, where NV is the number of synapses per unit volume, NA is the number of synaptic junctions per unit area of an electron micrograph, and d is the mean length of densities associated with the synaptic junctions. The profiles of synapses were marked on each micrograph of a set: a synapse was only marked if the synaptic junction was apparent and if at least two synaptic vesicles were seen in the presynaptic component of the synapse. Next, the lengths of densities of the synaptic junctions were measured using a ×10 magnifying lens with a graticule calibrated in 0.1 mm increments. If the synaptic junction profile appeared curved, the length of the junction was measured between its two ends. After measuring the length of the junction (d), the number of synapses per unit area (N_**A**_) were determined (23).

### Immunofluorescence Staining

For immunofluorescence, the frozen section of the brain tissues were fixed in 4% paraformaldehyde (PFA) overnight. After the brain tissues were fixed on the slicer table with OTC, multiple sets of frozen sections (20 μm thick, starting +2 mm to bregma and extending back to −3 mm to bregma) were cut in a cryostat (Leica CM1950) at −24°C, collected every three slicers on each SuperFrost Plus slide, and then stored at −80°C for further staining. For immunofluorescence staining, the brain slices were fixed with 4% paraformaldehyde for 10 min and blocked with 10% BSA for 60 min at room temperature (25, 26). The slides were incubated with primary antibodies of SYN (1:200, Abcam, MA, USA) and GAP-43 (1:200, Abcam) overnight at 4°C. After rinsing three times with PBS, the brain sections were incubated with the fluorescence-conjugated secondary antibodies (anti-mouse, 1:2,000) for 60 min at room temperature. The images were photographed using a confocal microscope (Leica TCS SP2). For the immunofluorescence staining analysis, four visual fields (400×) from each section of CA3 in the ipsilateral and contralateral areas were photographed under a confocal microscope (Leica TCS SP2), and the integrated optical density was analyzed using Image-Pro Plus 6.0 (Media Cybernetics Inc., MD, USA) designed to evaluate a given pixel, whether the red intensity (GAP-43 and SYN) was higher than the threshold values.

### Western Blot Analysis

The bilateral intact hippocampus tissue samples were collected and sonicated in a homogenizing buffer (RIPA with protease cocktail inhibitor, phosphatase inhibitor, and phenylmethanesulfonyl fluoride). Equal amounts of the samples were subjected to SDS-PAGE on 10% gel, and the proteins were transferred to a nitrocellulose membrane (GE Healthcare Life Sciences, PA, USA). The membrane was then incubated with the following primary antibodies overnight at 4°C: SYN (1:1,000; Abcam), GAP-43 (1:1,000; Abcam), and β-tubulin (1:1,000; Santa Cruz Biotechnology, TX, USA). After washing with TBST three times, the membrane was incubated with horseradish peroxide (HRP)-conjugated secondary antibodies for 60 min at room temperature and visualized by chemiluminescence (Pierce, IL, USA). The results were recorded with an imaging system (Bio-Rad, CA, USA) (27). The optical density ratio of the target band to β-tubulin served as the relative expression of the target protein.

### Statistical Analysis

All data were expressed as the mean ± SEM and analyzed using SPSS 16.0 (SPSS Inc., NY, USA) for parametric comparisons. The data of Western blot analysis, immunofluorescence staining, MWM test, and the number of synapses were analyzed using one-way analysis of variance followed by a Fisher's least significant difference *post hoc* test. The data of transmission electron microscopy, Western blot, and immunofluorescence staining comparing SH or EE ipsilateral vs. SH or EE contralateral were analyzed using *t*-test. The data of the correlation of cognitive function outcomes with GAP-43 and SYN were analyzed using Pearson's correlation coefficients. A probability value of <5% was considered to represent statistical significance.

## Results

### Cognitive Performance of the EE Group Improved in Acquisition Trial and Probe Test of MWM

**Figure 2:**
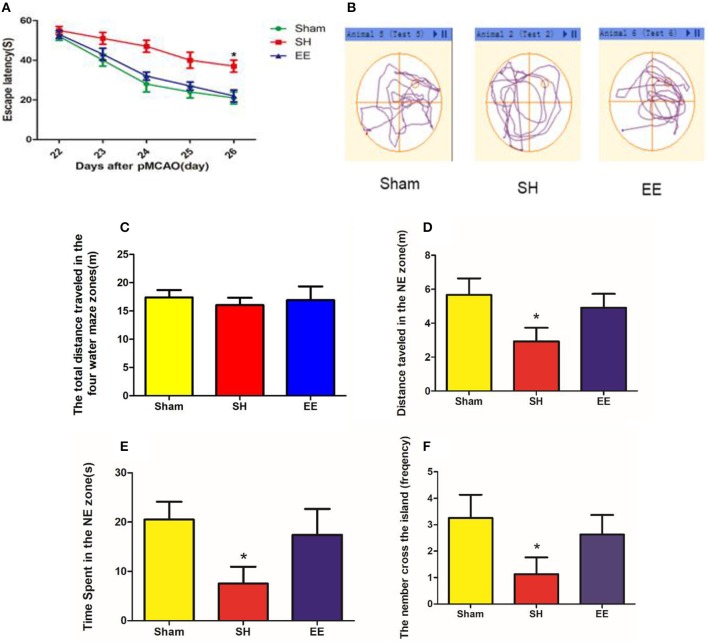
Cognitive performance after enriched environment (EE) intervention in the acquisition trial and probe test of Morris water maze (MWM). **(A)** Escape latency. Data are expressed as mean ± SEM, *n* = 8 per group. *P < 0.05, EE vs. the standard housing (SH) group. **(B)** Track plots showed the trace of mice in the tank during the probe test. **(C)** Total distance traveled in the four water maze zones. **(D)** Distance traveled in the correct (NE zone) quadrant. **(E)** Time spent in the correct quadrant (NE zone). **(F)** Number of crossings of the previous platform. Data are expressed as mean ± SEM, *n* = 8 per group. *P < 0.05, the EE group vs. the SH group.

The MWM test was performed to evaluate cognitive function in the sham, SH, and EE groups. Spatial acquisition trials were conducted on the first 5 days of cognitive evaluation. The results showed the change in escape latency from day 1 to 5 among different groups, and the EE group spent less time finding the platform compared with the SH group [Figure 2A, EE = 22.66 ± 3.22, SH = 40.26 ± 4.87, Sham = 21.10 ± 3.98(s), *P* = 0.012, *F*_(2, 21)_ = 3.46, *n* = 8]. The track plots showed the trace of mice in the tank during the probe test (Figure 2B). The probe test on the sixth day of cognitive evaluation revealed that the three groups had no significant difference in the total distance traveled in the four water maze zones [Figure 2C, EE = 17.76 ± 4.92, SH = 16.39 ± 3.67, Sham = 18.69 ± 3.78 (m), *P* = 0.692, *F*_(2, 21)_ = 0.081, *n* = 8]. The results revealed that the EE group traveled a significantly longer distance [Figure 2D, EE = 5.24 ± 1.31, SH = 3.16 ± 1.18, Sham = 5.74 ± 1.18 (m), *P* = 0.021, *F*_(2, 21)_ = 2.993, *n* = 8], spent more time [Figure 2E, EE = 18.94 ± 5.86, SH = 8.26 ± 4.36, Sham = 22.14 ± 4.68(s)*, P* = 0.009, *F*_(2, 21)_ = 4.291, *n* = 8] in the correct quadrant (NE zone), and crossed the former platform for more number of times [Figure 2F, EE = 18.94 ± 5.86, SH = 8.26 ± 4.36, Sham = 22.14 ± 4.68 (s), *P* = 0.007, *F*_(2, 21)_ = 5.041, *n* = 8] compared with the SH group.

### EE Improved the Number of Ipsilateral and Contralateral Synapses After pMCAO

**Figure 3:**
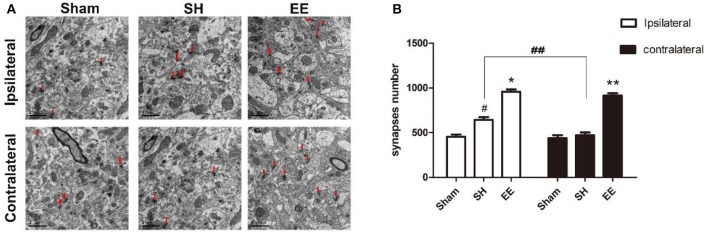
Enriched environment (EE) improved the number of ipsilateral and contralateral synapses after permanent middle cerebral artery occlusion (pMCAO). **(A)** Electron microscopy images of hippocampus CA3 showing the ipsilateral and the contralateral synapses in the sham, standard housing (SH), and EE groups. **(B)** Bar graph indicates the number of bilateral synapses in the sham, SH, and EE groups. Data are expressed as mean ± SEM, *n* = 8 per group. *P < 0.05, the EE group vs. the SH group in the ipsilateral. ^#^*P* < 0.05, the SH group vs. the sham group in the ipsilateral. **P < 0.05, the EE group vs. the SH group in the contralateral. ^##^*P* < 0.05, ipsilateral vs. contralateral in SH group.

The synapse change, which was related to cognitive function, was detected using transmission electron microscopy. Electron microscopy images indicated that the number of ipsilateral and contralateral synapses increased in the EE group when compared with the SH group [Figures 3A,B, ipsilateral EE = 989.87 ± 18.65, SH = 654.96 ± 21.65, Sham = 462.89 ± 18.99, *P* < 0.00, *F*_(2, 21)_ = 15.35, *n* = 8; contralateral EE = 979.76 ± 19.23, SH = 489.96 ± 21.65, Sham = 466.02 ± 22.41, *P* < 0.00, *F*_(2, 21)_ = 17.49, *n* = 8]. The number of synapses in the EE group showed no significant differences between ipsilateral and contralateral hemispheres [Figure 3B, ipsilateral EE = 989.87 ± 18.65; contralateral EE = 979.76 ± 19.23, *P* = 0.248, *t*_(15)_ = −1.079, *n* = 8], while the number of synapses in the ipsilateral hemisphere showed a significant upregulation than in the contralateral hemisphere in the SH group [Figure 3B, ipsilateral SH = 654.96 ± 21.65; contralateral SH = 489.96 ± 21.65, *P* = 0.018, *t*_(15)_ = 2.612, *n* = 8].

### EE Improved the Expression of Ipsilateral and Contralateral SYN After pMCAO

**Figure 4:**
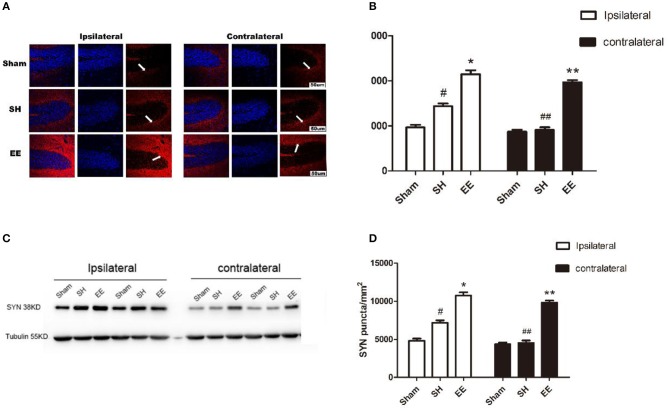
Enriched environment (EE) improved the expression of ipsilateral and contralateral SYN after permanent middle cerebral artery occlusion (pMCAO). **(A)** Representative immunofluorescence images of hippocampus CA3 of the ipsilateral and contralateral SYN (red) in the sham, standard housing (SH), and EE groups. Scale bar = 100 μm. **(B)** Bar graph shows the immunofluorescence quantification of the expression of ipsilateral and contralateral SYN among different groups. **(C)** Representative Western blot images of the ipsilateral and contralateral SYN in the sham, SH, and EE group. **(D)** Bar graph shows the Western blot quantification of the expression of ipsilateral and contralateral SYN among different groups. Data are expressed as mean ± SEM, *n* = 8 per group. *P < 0.05, the EE group vs. the SH group in the ipsilateral. ^#^*P* < 0.05, the SH group vs. the sham group in the ipsilateral. **P < 0.05, the EE group vs. the SH group in the contralateral. ^##^*P* < 0.05, ipsilateral vs. contralateral in the SH group.

Immunofluorescence staining and Western blot analysis were performed to quantify the expression of SYN on the synaptic vesicles, which could reflect the synapse function. Both the immunofluorescence images (CA3) and Western blot analysis showed that the expression of ipsilateral and contralateral SYN increased in the EE group compared with the SH group [Figure 4B, ipsilateral EE = 11,268.48 ± 198.95, SH = 7,874.06 ± 191.75, Sham = 4,798.19 ± 123.72, *P* < 0.00, *F*_(2, 21)_ = 47.97, *n* = 8; contralateral EE = 10,479.37 ± 98.76, SH = 4,816.06 ± 101.82, Sham = 4,881.02 ± 130.84, *P* < 0.00, *F*_(2, 21)_ = 45.95, *n* = 8; Figure 4D, ipsilateral EE = 0.915 ± 0.075, SH = 0.659 ± 0.031, Sham = 0.419 ± 0.022, *P* < 0.00, *F*_(2, 21)_ = 57.74, *n* = 8; contralateral EE = 0.837 ± 0.086, SH = 0.446 ± 0.082, Sham = 0.402 ± 0.094, *P* < 0.00, *F*_(2, 21)_ = 43.08, *n* = 8]. The expression of ipsilateral SYN was significantly upregulated in the SH group compared with the sham group (Figures 4A–D, *P* < 0.00).

### EE Improved the Expression of Ipsilateral and Contralateral GAP-43 After pMCAO

**Figure 5:**
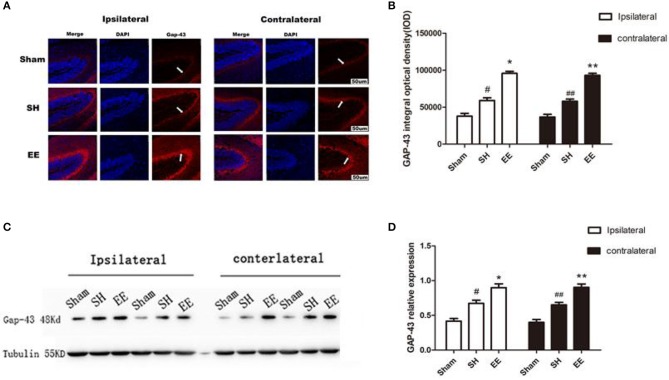
Enriched environment (EE) improved the expression of ipsilateral and contralateral GAP after permanent middle cerebral artery occlusion (pMCAO). **(A)** Representative immunofluorescence images of hippocampus CA3 of the ipsilateral and contralateral GAP (red) in the sham, standard housing (SH), and EE groups. Scale bar = 100 μm. **(B)** Bar graph shows the immunofluorescence quantification of the expression of ipsilateral and contralateral GAP-43 among different groups. **(C)** Representative Western blot images of the ipsilateral and contralateral GAP-43 in the sham, SH, and EE group. **(D)** Bar graph shows the Western blot quantification of the expression of ipsilateral and contralateral SYN among different groups. Data are expressed as mean ± SEM, *n* = 8 per group. *P < 0.05, the EE group vs. the SH group in the ipsilateral. ^#^*P* < 0.05, the SH group vs. the sham group in the ipsilateral. **P < 0.05, the EE group vs. the SH group in the contralateral. ^##^*P* < 0.05, the SH group vs. the sham group in the contralateral.

Whether the functional improvement in the EE group was due to increased neural plasticity was investigated by quantifying the expression of GAP-43. Both the immunofluorescence images (Ca3) and Western blot analysis showed that the expression of bilateral GAP-43 increased in the EE group compared with the SH group [Figure 5B, ipsilateral EE = 9,587.23 ± 78.95, SH = 6,514.91 ± 101.25, Sham = 4,232.19 ± 128.29; *P* < 0.00, *F*_(2, 21)_ = 42.07, *n* = 8; contralateral EE = 9,219.56 ± 93.33, SH = 6,869.96 ± 121.35, Sham = 4,166.42 ± 120.91. *P*< *0.00, F*_(2, 21)_ = *37.04, n* = *8*; Figure 5D, ipsilateral EE = 0.875 ± 0.073, SH = 0.696 ± 0.069, Sham = 0.441 ± 0.042, *P* < 0.00, *F*_(2, 21)_ = 31.74, *n* = 8; contralateral EE = 0.891 ± 0.084, SH = 0.642 ± 0.069, Sham = 0.432 ± 0.078, *P* < 0.00*, F*_(2, 21)_ = 33.28*, n* = 8]. It was interesting to find that the expression of bilateral GAP-43 was also significantly promoted in the SH group compared with the sham group (Figures 5A–D, *P* < 0.05).

### Correlation of Cognitive Function Outcomes With the Expression of GAP-43 and SYN in Bilateral Hemispheres

**Figure 6:**
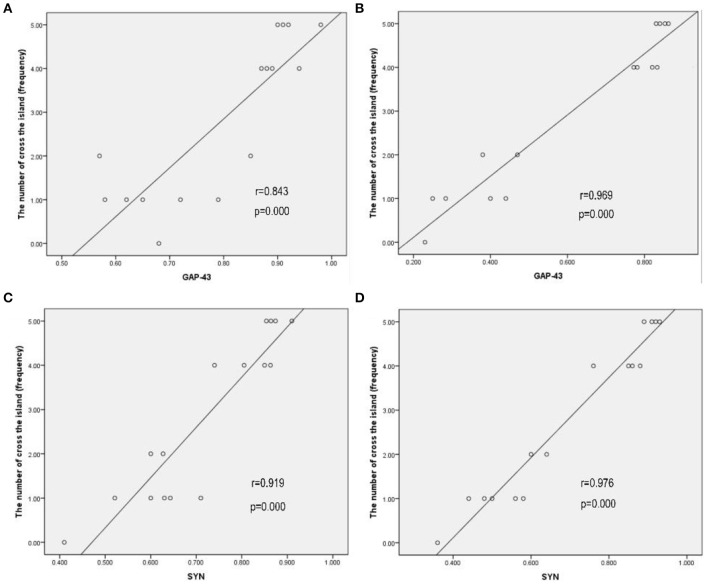
Cognitive function outcomes positively correlated with the relative expression of GAP-43 and SYN in bilateral hemispheres, and the correlation in the contralateral hemisphere was stronger than that in the ipsilateral hemisphere. **(A)** GAP-43, ipsilateral, *r* = 0.843. **(B)** GAP-43, contralateral, *r* = 0.969. **(C)** SYN, ipsilateral, *r* = 0.919. **(D)** SYN, contralateral, *r* = 0.976.

A possible correlation between cognitive function outcomes and the expression of GAP-43 and SYN in bilateral hemispheres was assessed by comparing the cognitive function (the number of crossings of the previous platform) with the relative expression of GAP-43 and SYN by using Pearson's correlation coefficients (r). The correlations between the cognitive function and relative expression of GAP-43 and SYN are shown in Figure 6. The cognitive function outcomes positively correlated with the relative expression of GAP-43 and SYN in bilateral hemispheres, and the correlation was stronger in the contralateral hemisphere than in the ipsilateral hemisphere (Figures 6A–D).

## Discussion

The present study revealed that EE could promote cognitive function after pMCAO. The MWM test results showed that the spatial memory and learning ability was significantly better in the EE group than those in the SH group.

The electron microscopy showed that EE improved the number of ipsilateral synapses after pMCAO compared with the SH group. The number of ipsilateral synapses was significantly higher in the SH group than in the sham group. Interestingly, a significant difference in the number of contralateral synapses was found between the EE and SH groups, but no difference was observed between the SH and sham groups. EE increased the number of contralateral synapses compared with the SH group. This finding suggested that the increase in the number of bilateral synapses, especially the contralateral synapses, might play an important role in cognitive recovery.

Synaptogenesis is considered a necessary mechanism for learning and memory. SYN is an important marker associated with synaptogenesis. A previous study showed that the presynaptic protein (SYN) was involved in hippocampus-dependent cognition after ischemic stroke (28). It is noteworthy that SYN knockout mice exhibited impaired spatial learning ability and memory loss without limb function impairment (29). Therefore, immunostaining and Western blot analysis were performed to detect the expression of SYN as an indicator of cognitive function recovery. The expression of SYN was found to be increased in the ipsilateral hemisphere 21 days after pMCAO in the SH group compared with the sham group. The expression of SYN in the ipsilateral hemisphere was significantly upregulated in the EE group compared with the SH group. However, in the contralateral hemisphere, no difference was found in the expression of SYN between the SH and sham groups. Furthermore, the EE group had increased the expression of SYN compared with the SH and sham groups. The results showed that the EE intervention could significantly upregulate the expression of SYN in the bilateral hemispheres. Also, the expression of SYN increased in the ipsilateral hemisphere in the SH group.

Neurons can extend axon branches and form new connections after injury (30). A previous study showed that the expression of GAP-43 increased during axon sprouting (31). GAP-43 immunostaining was used as a surrogate measure of axon growth and/or terminal sprouting in stroke models (2, 32). The expression of GAP-43 was detected by immunostaining and Western blot analysis to determine the axon growth in the chronic phrase after ischemia. The results revealed that the expression of GAP-43 in bilateral hemispheres increased 21 days after pMCAO in both EE and SH groups. The expression of GAP-43 was promoted in bilateral hemispheres in the EE group compared with the SH group.

Furthermore, the correlation of cognitive function outcomes with the relative expression of GAP-43 and SYN in bilateral hemispheres was analyzed. The cognitive function outcomes positively correlated with the relative expression of GAP-43 and SYN, and the correlation in the contralateral hemisphere was stronger than that in the ipsilateral hemisphere.

Previous studies demonstrated that the brain had an intrinsic capacity to compensate for tissue injury through remapping the survived networks (33). These processes are considered the foundation of spontaneous functional recovery after stroke (34). Various studies focused on the promotion of function in damaged areas or nearby regions (35, 36). Other study targets included both local injured and remote non-injured areas (37, 38). Some studies confirmed that stroke affected the interactions between hemispheres and changed biosignaling substances imparted by one hemisphere onto the other (39). However, the function of the contralateral hemisphere after stroke is controversial (34, 37). Some studies suggested that the contralateral hemisphere plays an active role in poststroke recovery (40, 41), while others considered that the contralateral hemisphere is a hindrance in poststroke recovery (42, 43). This study examined the expression of synaptophysin-related proteins in bilateral hemispheres poststroke after EE intervention on the basis of previous findings (44).

The expression of GAP-43 and SYN and the analysis of the correlation of cognitive function outcomes with the relative expression of GAP-43 and SYN in bilateral hemispheres showed that EE could improve the expression of GAP-43 and SYN in bilateral hemispheres. Also, the contralateral hemisphere might play a more important role in cognitive recovery after cerebral ischemia in mice. Furthermore, transmission electron microscopy showed that EE increased the number of synapses in the hippocampal CA1 3 weeks after pMCAO. Therefore, EE not only increased the expression of synaptic remodeling-related proteins but also increased the number of functional synapses in the bilateral hippocampus.

The limitation of this study was that the effects of ipsilateral synapses on the cognitive function could not be eliminated; therefore, the effects of the contralateral synapses alone could not be studied. The effects of each hemisphere on functional recovery in mice with secondary stroke may be observed through ipsilateral or contralateral infarctions. Moreover, the enriched sham group was not included in our study, and the effect found in the enriched lesioned animals should be explained in a cautious manner. This is because the effect might be a pure effect of the lesion, or the EE *per se*, and also it might be a combined effect of both EE and lesion. In our future study, the enriched sham group will be added for a better analysis.

Collectively, the present study demonstrated that EE could promote cognitive recovery after stroke *via* synaptic remodeling. The contralateral and ipsilateral synaptic remodeling affected the cognitive function. Moreover, the contralateral synaptic remodeling might play a more important role in cognitive recovery. Possible mechanisms might be the compensation for an ipsilateral function or the development of a favorable bilateral interaction. The actual underlying mechanism needs further investigation.

## Data Availability Statement

The datasets generated for this study can be found in the Med-X research institute of Jiaotong University, China.

## Ethics Statement

The animal study was reviewed and approved by The Institutional Animal Care and Use Committee of Fudan University approved the experimental protocols. The ethical agreement number is 20160858A232. Written informed consent was obtained from the owners for the participation of their animals in this study.

## Author Contributions

CW and QZ analyzed data and wrote the paper. KY revised the manuscript for important intellectual content. XS participated in electron microscopy and Western blot assay. YW and JW presented the experimental design and technical guidance.

### Conflict of Interest

The authors declare that the research was conducted in the absence of any commercial or financial relationships that could be construed as a potential conflict of interest.
